# Computational simulation of the flow dynamic field in a porous ureteric stent

**DOI:** 10.1007/s11517-022-02620-1

**Published:** 2022-06-28

**Authors:** Xiaohan Yang, Ali Mosayyebi, Dario Carugo

**Affiliations:** 1 Department of Mechanical Engineering, Faculty of Engineering and Physical Sciences, University of Southampton, Southampton, UK; 2 Department of Pharmaceutics, UCL School of Pharmacy, University College London, London, UK

**Keywords:** Ureteric stent, Porous material, Computational fluid dynamics, Ureteral obstruction

## Abstract

Ureteric stents are employed clinically to manage urinary obstructions or other pathological conditions. Stents made of porous and biodegradable materials have gained increasing interest, because of their excellent biocompatibility and the potential for overcoming the so-called ‘forgotten stent syndrome’. However, there is very limited characterisation of their flow dynamic performance. In this study, a CFD model of the occluded and unoccluded urinary tract was developed to investigate the urinary flow dynamics in the presence of a porous ureteric stent. With increasing the permeability of the porous material (i.e., from 10^−18^ to 10^−10^ m^2^) both the total mass flow rate through the ureter and the average fluid velocity within the stent increased. In the unoccluded ureter, the total mass flow rate increased of 7.7% when a porous stent with permeability of 10^−10^ m^2^ was employed instead of an unporous stent. Drainage performance further improved in the presence of a ureteral occlusion, with the porous stent resulting in 10.2% greater mass flow rate compared to the unporous stent. Findings from this study provide fundamental insights into the flow performance of porous ureteric stents, with potential utility in the development pipeline of these medical devices.

**Graphical abstract:**

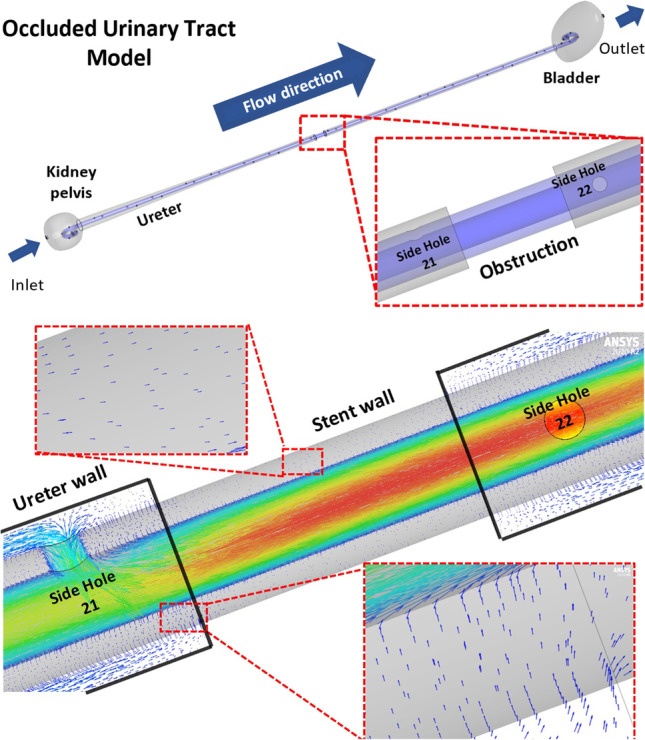

**Supplementary Information:**

The online version contains supplementary material available at 10.1007/s11517-022-02620-1.

## Introduction

Obstructive uropathy is a pathological condition whereby the urinary flow is impaired by obstructions, such as kidney stones or tumours [1, 2]. According to the National Health Service (NHS), more than 10% of the UK population suffered from kidney stones in 2019 [3]. Obstructions can occur in different regions of the urinary tract, especially at the junction between kidney pelvis and ureter (known as ureteropelvic junction, or UPJ) and between ureter and bladder (known as vesicoureteric junction, or VUJ) [1, 4]. A ureteric stent is a medical device consisting of a hollow flexible tube with side holes along its length [5], and is inserted within the ureter to maintain urinary drainage in the management of obstructive uropathy [6]. The most commonly used stent, known as ‘double-J’ stent, was invented by Finney in 1978 [6, 7]. Despite their proven and widespread clinical utility [8], stents often suffer from the formation of bacterial biofilms and encrustation over their surface, which can potentially cause stent failure. This in turn can negatively impact on a patient’s quality of life and may require stent replacement through surgical intervention [5, 9–12].

Several strategies have been implemented to address these causes of stent failure, through the development of novel stent architectures, constitutive materials, or surface coatings [13, 14]. Alternative designs to the traditional double-J stent have been introduced, including grooved or spiral-shaped stents to enhance urinary drainage [6, 15–17], and tail or dual-durometer stents to decrease irritative symptoms and pain for patients [6, 18, 19]. Surface modifications or coatings have also been applied on stents, with the aim of reducing formation of biofilms and encrustation [14, 20, 21]. Moreover, drug-eluting coatings have been devised to enable controlled release of anticancer, anti-inflammatory, or antimicrobial compounds (such as triclosan [22], tigecycline and rifampicin [23]). This latter approach has proven to be effective in limiting fibroproliferative reactions, inhibiting bacterial adhesion, and reducing formation of biofilms and encrustation [20, 24, 25]. Another important area of stent design that has been the focus of engineers and scientists, is the one of stent’s constitutive materials [14]. Metals, inert polymers, and biodegradable materials are the three main types of substrates used in stent manufacturing [5, 6, 13, 21]. Biodegradable materials have been investigated more recently because of their excellent biocompatibility [25]. Moreover, their porous structure can be permeated by biological fluids, can act as a reservoir for the release of active pharmaceutical ingredients [26, 27], or can degrade over time thus avoiding the need for stent removal through surgical intervention [5, 28, 29].

A challenge associated with biodegradable stents is to control the material degradation process, which is dependent upon material properties (including porosity and permeability), chemical composition, mechanical loading, and fluid flow [30]. Biofilms and encrustation could also form on degraded fragments of the stent, which may potentially lead to urinary tract blockage [31, 32]. Notably, achieving uniform stent degradation and control over the size of degraded fragments is a challenge that should be addressed to enable widespread clinical translation of innovative biodegradable stents.

Computational fluid dynamic (CFD) simulations are a powerful tool to investigate the flow dynamic field and drainage performance of ureteric stents [33–36]. Recent studies have employed CFD modelling to evaluate the influence of stent architecture and ureter anatomy on urinary drainage, and to identify regions of the stent that are more likely to suffer from encrustation or particle accumulation [9, 33, 36–41]. However, to the best of the authors’ knowledge, no previous study has investigated the flow field in the urinary system in the presence of a porous ureteric stent. The latter is particularly important for biodegradable and/or drug-eluting stents, as flow can modulate the rate and uniformity of stent degradation as well as drug release kinetics. In order to address this research gap, the present study aimed at evaluating the drainage performance of porous ureteric stents by designing a CFD model of the stented urinary tract. The effects of material’s permeability, presence of a complete ureteral occlusion, and presence of side holes along the stent were evaluated. Overall, findings from this study provide fundamental insights into the flow performance of porous ureteric stents, with potential utility in the development pipeline of these medical devices.

## Methods

### Design of the urinary system and stent model geometries

**Fig. 1:**
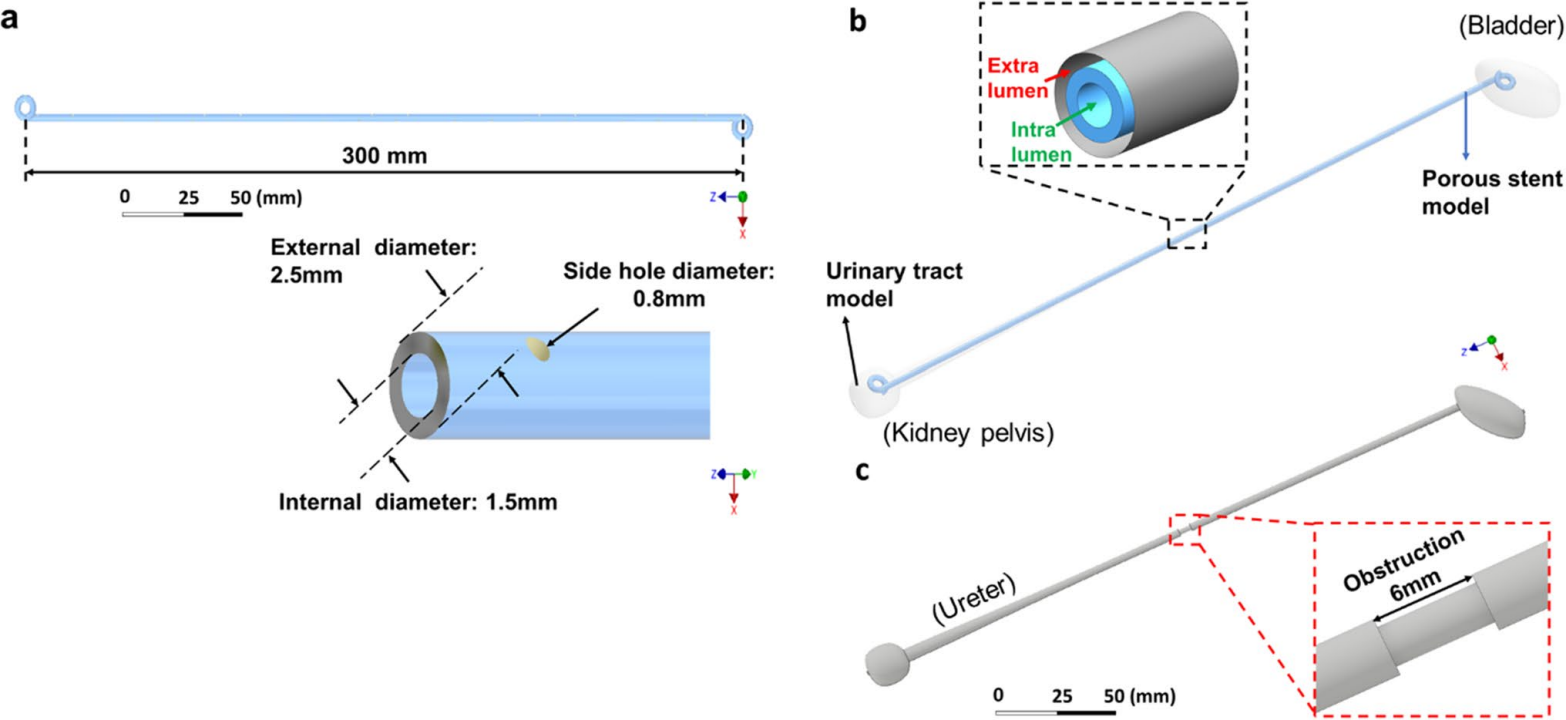
**a. **Graphical representation of the stent geometry, with indicated key dimensional properties, such as total length, internal and external diameter, and side hole diameter. **b** Graphical representation of the stented urinary tract model. The inset shows a zoomed-in view of the model, with indicated the internal (intra lumen) and external (extra lumen) compartments of the stent. **c** Occluded urinary tract model. The inset shows a zoomed-in view of the 6 mm long, obstructed region of the middle ureter

Computer-aided design (CAD) drawings of the urinary system and porous stent models were built in Inventor Pro 2021 (Autodesk®, USA). The stent geometry was reconstructed from a commercially available double-J stent (Universa®, Cook® Medical, USA), with a total of 42 side holes having a diameter of 0.8 mm. Four of these side holes were located at each coil of the stent. The inner and outer diameters of the stent were equal to 1.5 mm and 2.5 mm, respectively. A graphical representation of the stent geometry, including key dimensional values, is shown in Fig. 1a. The urinary tract model geometry was designed based on ex vivo data collected from pig ureters, as described in a previous study [42]. The model included three main compartments, e.g. kidney pelvis, ureter and bladder, and was subsequently coupled with the stent model (as shown in Fig. 1b). Concerning the ureter model, its diameter decreased from approximately 6.0 to 3.0 mm in the proximal segment, and remained almost constant and equal to approximately 2.5 and 2.4 mm in the middle and distal segments, respectively. It then slightly increased to 2.9 mm at the VUJ. The obstructed urinary tract model comprised a complete constriction of the middle ureter, as shown in Fig. 1c, which was placed between side hole 21 and side hole 22 (note that a fillet was not built into the model at the obstructed region). Notably, clinical data showed that 40% of mid-ureter stones cannot be spontaneously passaged [43] and previous numerical studies of the flow dynamics in the stenosed ureter often modelled a constriction of the middle ureter [37, 39], as for the present study.

### Construction of the numerical mesh

The computational fluid dynamic simulations in this study were carried out using Ansys 2020 R1 software package (Ansys Inc., USA), which included Space Claim, Fluent 2020 R1, and CFD-Post 2020 R1. Upon generation of the model design in Autodesk® Inventor Pro 2021, Space Claim was employed to patch side hole surfaces (to allow subsequent calculation of the fluid flow rate through each side hole) and to label each surface of the urinary system. The model geometry was subsequently discretised into polyhedral mesh volumes, using Fluent 2020 R1. Notably, previous CFD studies comparing polyhedral and tetrahedral meshing have shown that the former provides improved computational convergence and the ability to resolve flow metrics nearby surfaces with greater homogeneity [44]. The mesh growth rate, which is the increase in element edge length with each succeeding layer of elements, was set to 1.2. The curvature normal angle, which is the maximum allowable angle that one element edge is allowed to span, was instead set to 14°. Considering the dimensions of the stented urinary system model and the meshing time, the maximum and minimum cell size were set to 3 mm and 0.2 mm, respectively. In order to resolve the flow field through side holes of the stent, the proximity and curvature size functions were employed to generate a finer mesh in proximity to side holes. To obtain an accurate determination of flow rate and fluid velocity at the stent surfaces, local mesh sizing (of 0.1 mm) was set over these regions. During meshing, all stent surfaces were also set as ‘internal’. Three and one boundary layers were applied to the stent and urinary tract surfaces, respectively. Finally, in order to improve the mesh quality, the ‘improve volume mesh’ function was used and the cell quality limit set to 0.15. A mesh sensitivity analysis was carried out to identify the appropriate meshing parameters, that represented an effective compromise between solution accuracy and computational cost. The results of this analysis are reported in the Supplementary Material section (Figures S1-S3), and the total number of mesh cells for the selected mesh was equal to 5 528 205 (unoccluded stented model) and 5 506 567 (occluded stented model).

### Flow regime and governing equations

The Reynolds number (Re) is a dimensionless quantity that can be used to predict the flow regime in a given system. It is defined as the ratio of inertial forces to viscous forces within a fluid, as shown in Eq. 1 below.

1 $$Re=\frac{\rho uL}{\mu }$$

where *ρ* is the density of the fluid, *u* is the mean fluid velocity, *μ* is the dynamic viscosity of the fluid, and *L* is a characteristic linear dimension (corresponding to the hydraulic diameter of a conduit in pipe flow). A previous study by Kim and co-authors has estimated that the Reynolds number in the ureter varies from 6.21 to 30.42 [39]. In their study, the ureter model was constructed by averaging ureteral lengths and diameters from 19 men, obtained from two-dimensional axial computed tomography (CT) data of patients who did not have any clinical history of urological diseases [37]. Fluid physical properties were set to those of healthy urine. It should also be noted that earlier studies have indicated that there are no significant age and gender differences in ureteral diameter [45]. Given that for Reynolds numbers < 2300 the flow regime can be typically regarded as laminar [46], urinary flow in the stented ureter model was also assumed to be laminar herein.

Moreover, in the present study, urine was assumed to be a continuum, incompressible, and Newtonian liquid. The urine flow field was thus determined by solving for mass (Eq. 2) and momentum (Eq. 3) conservation equations (referred to as Navier–Stokes equations).

2 $$\nabla \bullet \left(\overrightarrow{v}\right)=0$$

3 $$\frac{\partial \overrightarrow{v}}{\partial t}+\rho \overrightarrow{v}\bullet \nabla \overrightarrow{v}=-\nabla P+\mu {\nabla }^{2}\overrightarrow{v}$$

where $\overrightarrow{v}$ is the fluid velocity vector, *P* is the fluid pressure, and *t* is time.

The flow field within the porous medium (i.e., the stent wall) was characterised by the addition of a source term in the momentum conservation equation. For a homogeneous porous medium, this term is the sum of a viscous loss component and an inertial loss component (as shown in Eq. 4).

4 $${S}_{i}=-\left(\frac{\mu }{\alpha }{v}_{i}+C\frac{1}{2}\rho \left|v\right|{v}_{i}\right)$$

where *S*_*i*_ is the source term for the *i*^th^ (*x*, *y*, or *z*) momentum equation, and *α* and *C* represent the medium permeability and inertial resistance factor, respectively.

In the case of laminar flow through a porous medium, as it is the case in this study, inertial forces can be neglected given the low fluid velocity [47]. The fluid pressure drop can thus be considered to be proportional to the fluid velocity, and the inertial resistance factor (*C*, in Eq. 4) can be considered constant and equal to 0. Thus, the porous medium model reduces to Darcy’s law (shown in Eq. 5) [48].

5 $$\nabla P=-\frac{\mu }{\alpha }\overrightarrow{v}$$

### Model assumptions

Some key assumptions were made when implementing the CFD model in this study, as indicated below:

(i) The transfer of heat between the fluid, stent, and surrounding tissues was considered negligible.
(ii) The walls of ureter, bladder and kidney were considered rigid and stationary. This also implied neglecting the peristaltic activity of the ureter, consistently with previous studies that modelled the flow dynamics in the stented ureter either computationally or experimentally [36, 49]. Notably, in vivo observations have shown that ureteral stenting typically results in a pronounced reduction of peristalsis (particularly in the long-term) [50].
(iii) The porous medium was assumed to be isotropic and homogeneous.
(iv) The fluid was assumed to be Newtonian and incompressible, which is also consistent with previous investigations of urinary flow in the stented ureter [9, 36].
(v) As described above, inertial forces were neglected (including inertial losses through the porous medium) and fluid flow was assumed laminar.

### Boundary conditions and solution methods

A pressure boundary condition was applied on the model inlet and outlet; constant gauge pressure values were set to 97.8 Pa (kidney pelvis) and 0 Pa (bladder), respectively, which is coherent with previous models of urinary flow dynamics [40]. A no-slip boundary condition was set at the bladder, ureter, and kidney walls, which is also consistent with earlier studies [9, 36]. A no-slip boundary condition was also applied at the inner and outer surfaces of the unporous stent. Concerning the porous stent wall, the numerical model uses and reports a superficial velocity inside the porous medium, based on the volumetric flow rate, to ensure continuity of the velocity vectors across the porous medium interface [48]. Urine has similar physical properties to water [34], thus density and dynamic viscosity of the working fluid were set to 997.044 kg/m^3^ and 0.001 Pa × s, respectively. In this study, permeability of the porous stent was varied in the range of 10^−18^ and 10^−10^ m^2^ (as outlined in Table 1); a permeability value of 0 corresponds to the unporous stent. Values of permeability were selected to encompass those of porous materials that are often employed to manufacture medical devices or scaffolds, such as collagen hydrogels (10^−18^–10^−0^ m^2^) [51], alginate hydrogels (10^−12^–10^−0^ m^2^) [52], and poly acrylamide gels (10^−19^–10^−17^ m^2^) [53]. The gravitational acceleration (*g*) was set to 9.8 m/s^2^ in the direction of *y* (i.e., oriented towards the ground), which corresponds to a patient lying down on their back. In a specific group of simulations, it was instead oriented in the direction of *z* to model a patient in standing position. The SIMPLE algorithm was selected as a pressure-based solution method, and gradients were computed using the least squares cell–based method. The solution was assumed to have reached convergence when residuals for all variables had reached a value of 10^−10^, which corresponded to approximately 1500 iterations.

**Table 1:** Simulation case studies that were modelled in this investigation. For each case study, the Table reports values of stent permeability (in m^2^), number of stent side holes, and whether or not the urinary tract model is obstructed

Case n	Stent permeability (m^2^)	Number of side holes	Ureteral obstruction
1	0	42	No
2	1 × 10^−10^	42	No
3	1 × 10^−11^	42	No
4	1 × 10^−12^	42	No
5	1 × 10^−14^	42	No
6	1 × 10^−18^	42	No
7	1 × 10^−10^	0	No
8	0	42	Yes
9	1 × 10^−10^	42	Yes

### Simulation case studies

A number of different simulations were carried out in this study, as outlined in Table 1. A group of simulations (from Case 1 to Case 6) were performed to investigate the effect of stent permeability on urinary drainage within the unobstructed urinary system. Another group of simulations (Case 2 and Case 7) were performed to evaluate the effect of side holes on the drainage performance of a porous stent. Finally, Case 8 and Case 9 were modelled to compare performance of porous and unporous stents, in the presence of a ureteral occlusion. In these last two groups of simulations, only the greatest stent permeability (10^−10^ m^2^) was considered for the porous stent, as it was associated with the most improved drainage performance (among values investigated in the present study). In all case studies reported in Table 1, the model was oriented horizontally (i.e., corresponding to a patient in supine position) as no detectable difference was found when comparing its flow dynamic field with the one of a vertically oriented model (i.e., corresponding to a patient in standing position).

### Determination of stent drainage performance

The stent flow dynamic performance was assessed by quantifying the fluid velocity magnitude at given positions along the model, the mass flow rate within the stent (intra lumen) and outside the stent (extra lumen), and the mass flow rate through each individual side hole. The mass flow rate ($\dot{m})$ was defined according to Eq. 6.

6 $$\dot{m}=\rho \bullet u\bullet A$$

where *A* is the cross-sectional area through which the mass flow rate was calculated and *u* is the mean fluid velocity over the cross-sectional area.

**Fig. 2:**
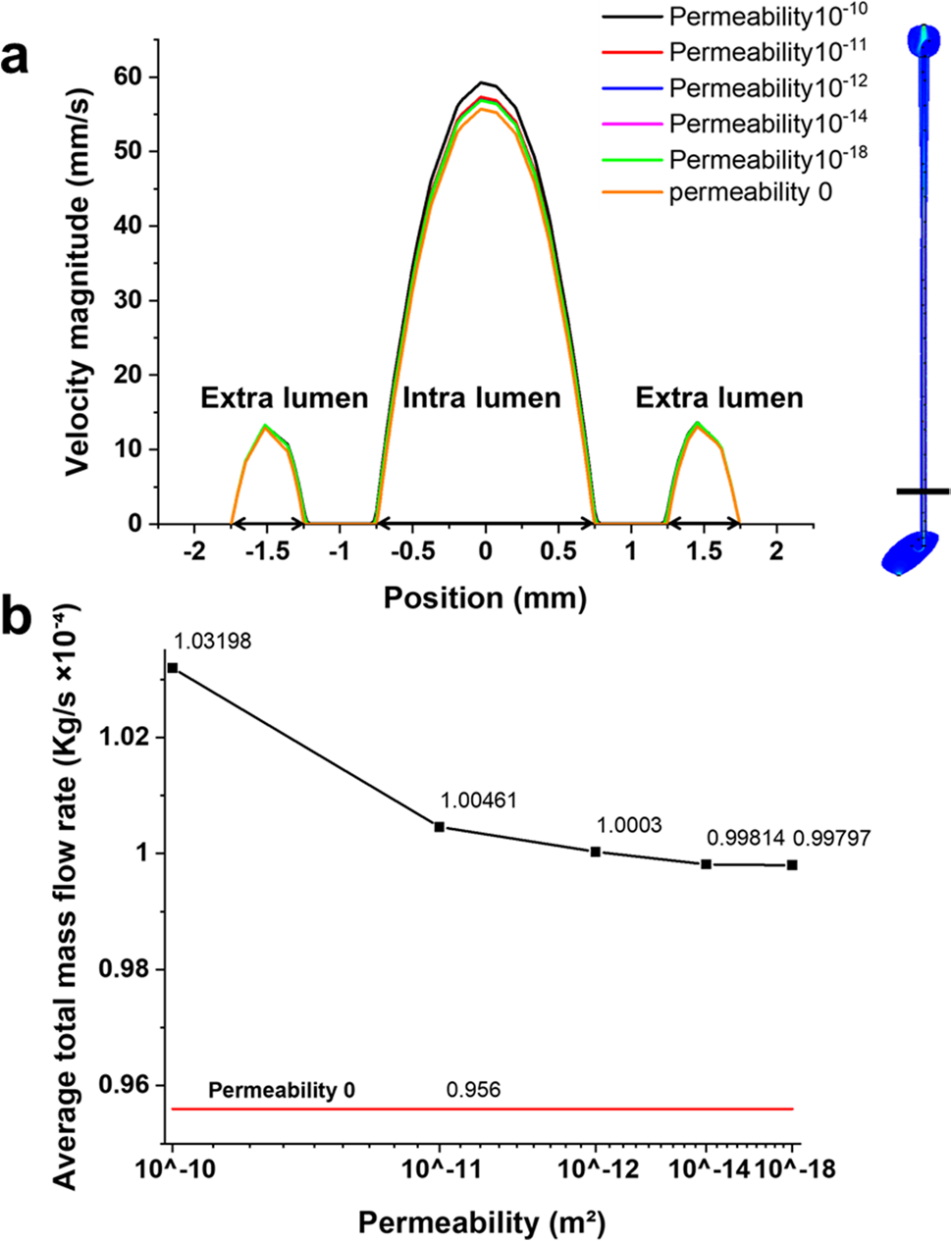
**a.** Profile of velocity magnitude (in mm/s) taken along a line located in proximity to the VUJ, between side hole 35 and side hole 36, for each value of stent permeability investigated (including the unporous stent). The different values of permeability investigated are reported in the figure legend. The model schematic on the right-hand side shows the location of the line along which the velocity values were taken. Intraluminal and extraluminal compartments are also labelled on the graph. **b** The average total mass flow rate (in kg/s) through the stented ureter model, determined numerically for stents of different permeability. Permeability values decrease from left to right (i.e., from 10^−10^ to 10^−18^ m^2^). The horizontal red line corresponds to the average total mass flow rate for the unporous stent (permeability: 0 m.^2^)

Surfaces were built in between each pair of side holes along the model, to determine the mass flow rate through the intraluminal and extraluminal compartments of the stent and the mass flow rate through the stent wall (Fig. 2b). For the porous stent, the total flow rate was defined according to Eq. 7. For the unporous stent, the flow rate through the constitutive material of the stent wall was equal to 0 kg/s, as in this case flow exchange between intraluminal and extraluminal compartments could only occur through side holes.

7 $${\dot{m}}_{T}={\dot{m}}_{\mathrm{Intra}}+{\dot{m}}_{\mathrm{Extra}}+{\dot{m}}_{\mathrm{Stent}}$$

where ${\dot{m}}_{T}$, ${\dot{m}}_{\mathrm{Intra}}$,${\dot{m}}_{\mathrm{Extra}}$, and ${\dot{m}}_{\mathrm{Stent}}$ correspond to the total mass flow rate, intraluminal mass flow rate, extraluminal mass flow rate, and mass flow rate through the porous stent wall, respectively. In some instances, the mass flow rate is reported as an average over multiple determinations along the model, to obtain a ‘global’ assessment of stent’s drainage performance.

## Results

This section describes the results obtained from the CFD simulations, which provide a characterisation of the flow performance of a porous stent within either an unobstructed or obstructed model of the ureter. Results are presented in the form of fluid velocity or mass flow rate determinations, at different regions of interest within the model.

As described earlier, steady-state simulations were performed by imposing a constant gauge pressure of 97.8 Pa at the kidney pelvis and 0 Pa at the bladder. An initial series of simulations were carried out to evaluate the effect of stent permeability on the urinary flow field. Permeability was decreased from 10^−10^ to 10^−18^ m^2^ (individual values assessed corresponded to 10^−10^, 10^−11^, 10^−12^, 10^−14^, and 10^−18^ m^2^), and results were compared with the unporous stent. Figure 2a shows the profile of velocity magnitude taken along a line located in proximity to the VUJ, between side hole 35 and side hole 36, for each value of stent permeability investigated. As it can be observed, fluid velocity within the stent (i.e., intraluminal velocity) is significantly greater compared to the fluid velocity outside the stent (i.e., extraluminal velocity). The porous stent with the greatest permeability (10^−10^ m^2^) showed the highest intraluminal velocity magnitude among all stents investigated, with a maximum velocity of 59.3 mm/s. When stent permeability was reduced to 10^−11^ m^2^, the maximum intraluminal velocity decreased to 57.3 mm/s. Reducing stent permeability further, only caused a marginal change in the velocity profile; i.e. stents with a permeability between 10^−12^ and 10^−18^ m^2^ showed a comparable maximum intraluminal velocity of approximately 56.9 mm/s. The unporous stent presented the lowest intraluminal velocity magnitude (55.6 mm/s), which was only marginally smaller than the porous stent with the lowest permeability (10^−18^ m^2^). Concerning the extraluminal velocity (Fig. 2a), all porous stents exhibited comparable values of velocity magnitude. The unporous stent showed only a marginal reduction in maximum extraluminal velocity (13.07 mm/s) compared to the porous stent with the greatest permeability (13.65 mm/s).

Simulations were also carried out to evaluate whether the velocity profile would vary as a consequence of changing the model’s orientation. Supplementary Figure S4 shows that there was no detectable change when the orientation was varied to model either a patient in supine or standing position, for both porous and unporous stents. This may suggest that the dynamic pressure gradients dominate over gravitational effects in determining the flow dynamic field within the stented ureter model. On the basis of these results, a fixed model orientation (corresponding to a patient in supine position) was adopted for all simulation case studies.

The average total mass flow rate through the ureter model is shown in Fig. 2b, for the different stent permeabilities investigated. When compared with the unporous stent, all types of porous stent presented a greater average mass flow rate through the ureter model. The average mass flow rate decreased with decreasing stent permeability (from left to right, in Fig. 2b), following a non-linear relationship. There was a 3.07% decrease in average mass flow rate with decreasing permeability from 10^−10^ to 10^−12^ m^2^. However, mass flow rate decreased only marginally when the permeability was decreased further from 10^−14^ to 10^−18^ m^2^.

**Fig. 3:**
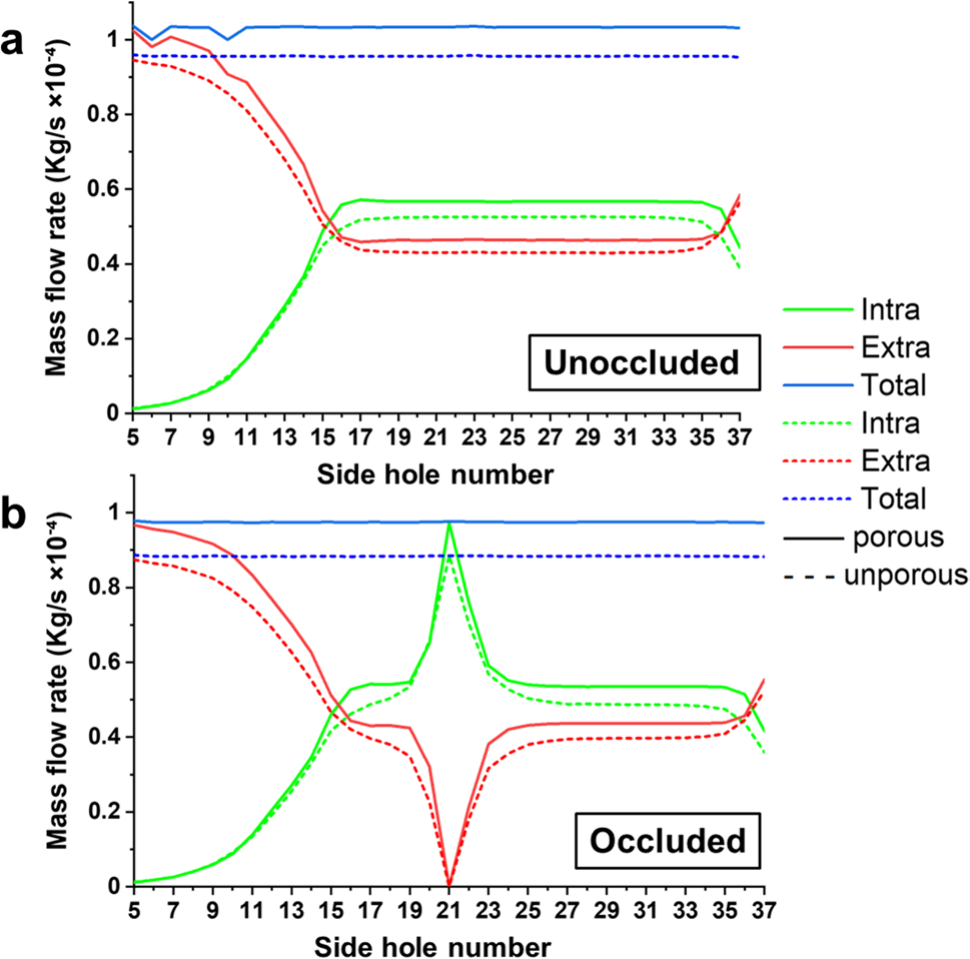
Total, intraluminal, and extraluminal mass flow rates (in kg/s) at different positions along the ureter (indicated by the side hole number on the *x-*axis), for both porous and unporous stents. Results are reported for both unoccluded (**a**) and occluded (**b**) ureter models. The porous stent has a permeability of 10^−10^ m^2^

Figure 3 shows the intraluminal, extraluminal, and total mass flow rate in the porous and unporous stented ureter, for both the unoccluded (Fig. 3a) and occluded (Fig. 3b) models. Dashed lines on the graph correspond to the unporous stent, whilst continuous lines correspond to the porous stent. Permeability of the porous stent was set to 10^−10^ m^2^ in these simulations, since this value resulted in the greatest average urinary flow rate compared to the other stent permeability values investigated (as shown above in Fig. 2b). Figure 3a shows that, in the unoccluded ureter, urinary flow in the proximal ureter was predominately localised within the extraluminal compartment of the stent. However, as the ureter diameter reduced from the proximal to the middle ureter, part of the urinary flow was directed into the stent; this resulted in a reduction of extraluminal flow rate and a corresponding increase in intraluminal flow rate (as shown in Fig. 3a). The flow rate distribution between intraluminal and extraluminal compartments remained almost unchanged in the remaining regions of the middle and distal ureter. As the stent entered the bladder compartment, the cross-sectional area of the urinary tract model significantly increased and the fluid started to exit the stent, which was reflected in a corresponding increase of extraluminal flow rate and a reduction of intraluminal flow rate. All mass flow rates evaluated (i.e., total, intraluminal, and extraluminal) were greater when the porous stent was employed instead of the unporous stent. The average total flow rate through the stented ureter increased of 7.6 × 10^−6^ kg/s, when the unporous stent was replaced with a porous stent of permeability 10^−10^ m^2^.

Similar considerations concerning the urinary flow distribution apply to the case of an occluded ureter model, as shown in Fig. 3b. The main difference between unoccluded and occluded models is observed in proximity to the ureteral occlusion, which was located between side hole 21 and side hole 22. Notably, the urinary flow was directed into the stent to by-pass the occlusion, and subsequently exited the stent after the occlusion. Given that a complete occlusion of the ureteral lumen was modelled in this study, the whole mass flow rate was localised within the stent lumen just prior to the occlusion (i.e., at side hole 21, the intraluminal flow rate is equal to the total flow rate). As for the unoccluded ureter model, the total, intraluminal, and extraluminal mass flow rates were all greater when the porous stent was employed instead of the unporous one. Moreover, the total flow rate in the occluded ureter model was lower than in the unoccluded model, and was equal to 9.75 × 10^−5^ kg/s (porous stent) and 8.84 × 10^−5^ kg/s (unporous stent).

**Fig. 4:**
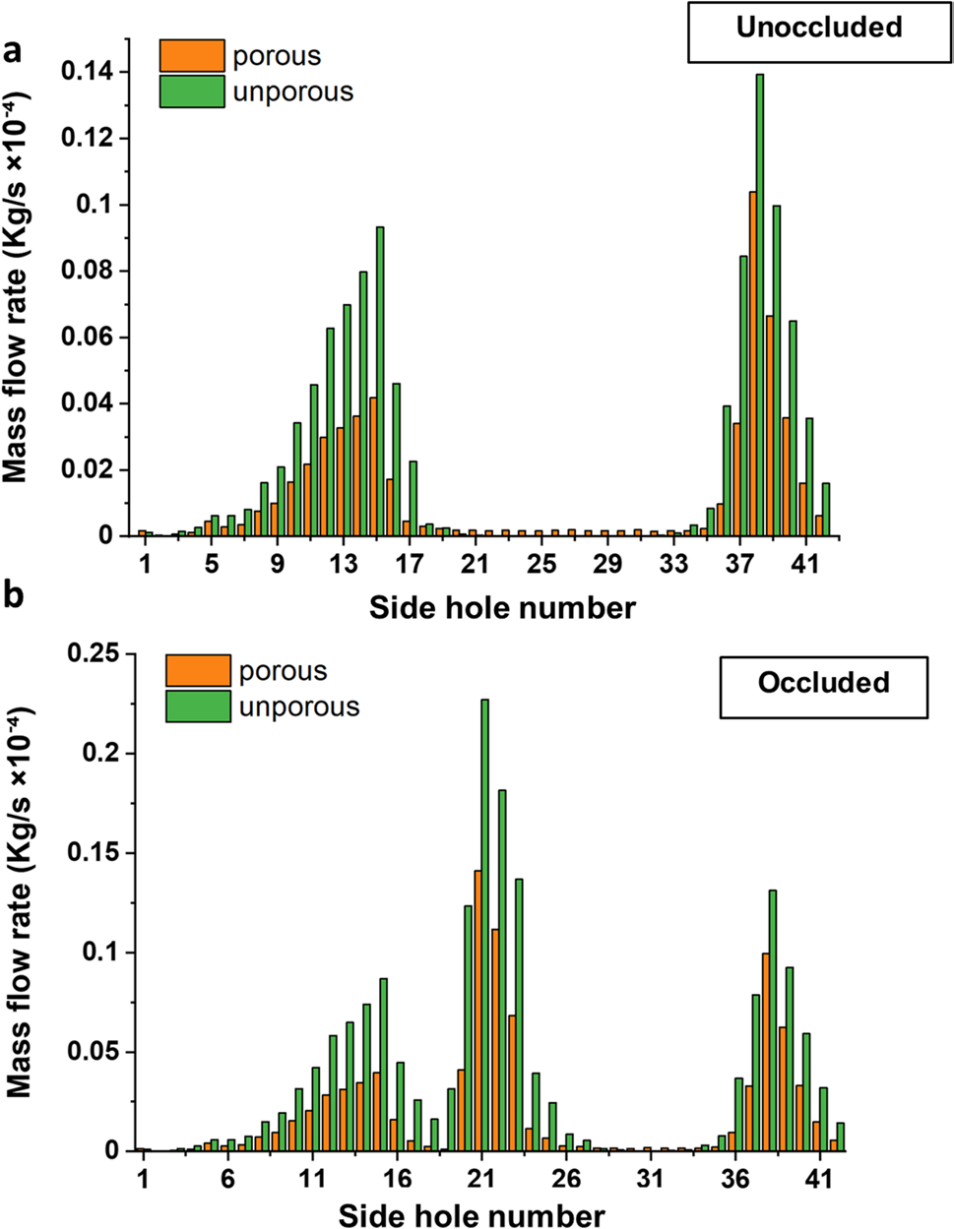
Mass flow rate (in kg/s) through individual side holes of the stent, for both unporous and porous stents. Results are reported for both **a** unoccluded and **b** occluded ureter models

The mass flow rate through each individual side hole of the stent is reported in Fig. 4 for both unporous stent and porous stent (permeability: 10^−10^ m^2^), either in the absence (Fig. 4a) or presence (Fig. 4b) of a ureteral occlusion. As it can be observed, only a proportion of side holes were characterised by appreciable levels of mass flow rate; these were referred to as ‘active’ side holes. In the unoccluded ureter model (Fig. 4a), these included (i) side holes located in the proximal region of the ureter, where a proportion of the extraluminal flow entered the stent as the ureter diameter gradually reduced, and (ii) side holes located in proximity or distal to the VUJ, as urine exited the stent in those regions of the urinary tract. In the presence of a ureteral obstruction (Fig. 4b), side holes located just nearby the occlusion—i.e., both proximally and distally to the occlusion—were also interested by flow exchange between intraluminal and extraluminal compartments. The greatest flow rate was indeed quantified at side hole 21 of the stent. Figure 4a also shows that, in the unoccluded ureter, the mass flow rate through side holes was generally 40–50% greater in the unporous stent compared to the porous stent. Interestingly, side holes located in the middle and distal ureter (from side hole 20 to 33) presented low but quantifiable levels of mass flow rate in the porous stent, which was instead absent in the same side holes of the unporous stent. Similar considerations apply to the obstructed ureter model (Fig. 4b), with the unporous stent overall showing greater mass flow rate values through side holes compared to the porous stent.

**Fig. 5:**
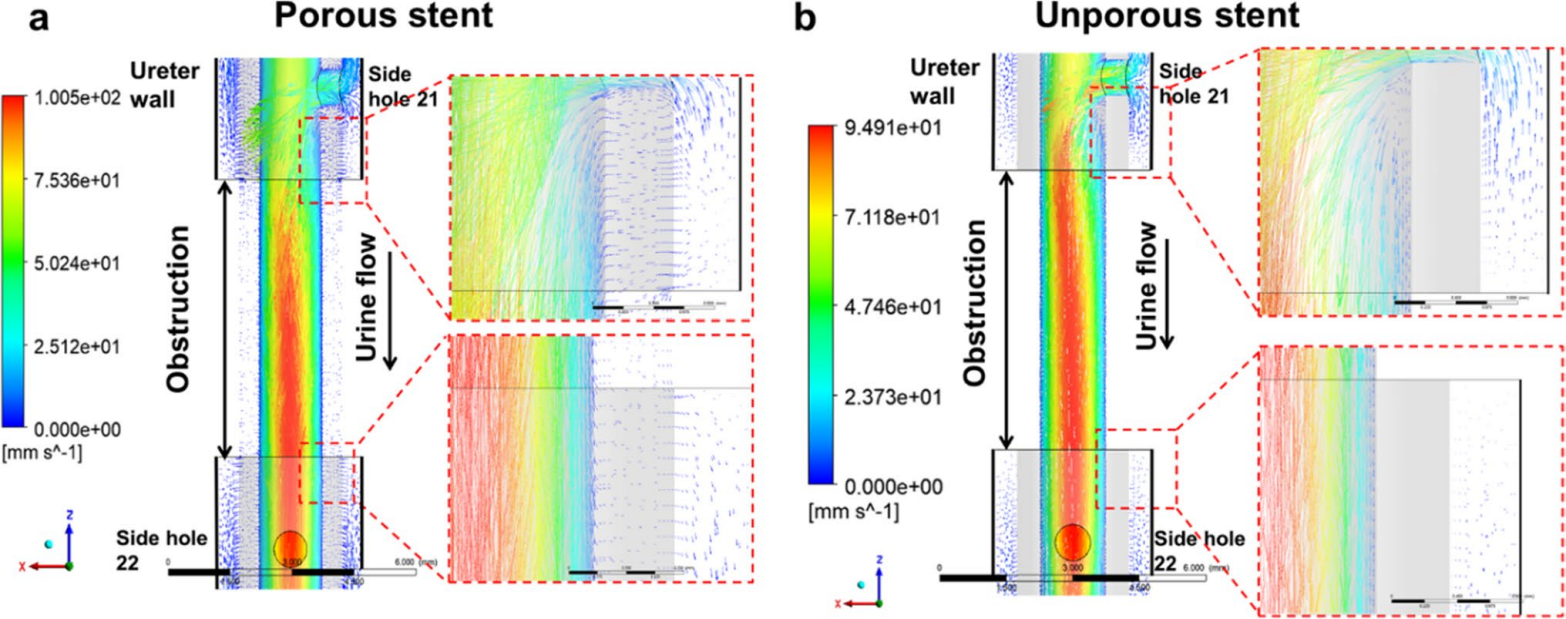
The fluid velocity vector field at the occluded region of the ureter model, taken over the mid-plane of the model (in the *y*-direction), for both porous (**a**) and unporous (**b**) stents. Vectors are coloured based on the velocity magnitude values (in mm/s) reported in the corresponding coloured bars. Zoomed-in views of the vector field are reported in the red dashed boxes, where the grey areas correspond to the stent wall

Figure 5 shows the fluid velocity vector field at the occluded region of the ureter model, taken over the mid-plane of the model (in the *y*-direction), and plotted for both porous (Fig. 5a) and unporous (Fig. 5b) stents. The porous stent had a permeability of 10^−10^ m^2^. The ureteral occlusion was located between side hole 21 and side hole 22, and the grey areas correspond to the stent wall. In the case of both porous and unporous stents, it can be observed that the flow was largely diverted into the stent at side hole 21, which is located proximally to the occlusion. The maximum fluid velocity over this plane was of 100.5 mm/s and 94.9 mm/s for the porous and unporous stents, respectively. The zoomed-in views of the vector field (see red dashed boxes in both Fig. 5a and Fig. 5b) show that − in the porous stent − a number of velocity vectors was directed across the stent wall (in particular nearby side holes 21 and 22), corresponding to fluid flowing from the extraluminal to the intraluminal compartments through the porous stent wall (Fig. 5a). As expected, there was no fluid motion through the wall of the unporous stent (Fig. 5b).

**Fig. 6:**
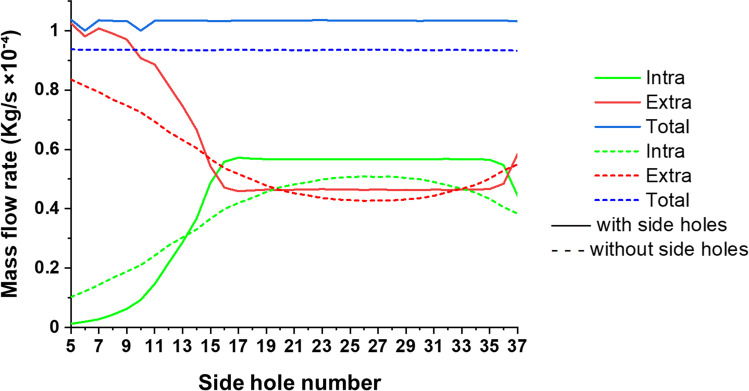
Total, intraluminal, and extraluminal mass flow rate at different positions along the unoccluded ureter model (indicated by the side hole number on the *x*-axis), for a porous stent with side holes and without side holes. In these simulations, the stent had a permeability of 10^−10^ m^2^

Figure 6 shows the total, intraluminal, and extraluminal mass flow rates along the unoccluded ureter model for two different designs of porous stent, i.e. containing side holes (continuous lines) and without side holes (dashed lines). The porous stent with side holes had a greater average total mass flow rate (1.03 × 10^−4^ kg/s) compared to the porous stent without side holes (9.35 × 10^−5^ kg/s). The main differences between the two stent designs were in the intraluminal flow rate in the proximal region of the ureter (i.e., until side hole 14), and in the extraluminal flow rate in the middle and distal ureter. As discussed previously, the stent containing side holes showed a significant increase in the intraluminal flow rate in the proximal region of the ureter (accompanied by a corresponding decrease in extraluminal flow rate), after which both intraluminal and extraluminal flow rates plateaued and remained approximately constant until the most distal region of the ureter where the intraluminal flow rate decreased (and conversely the extraluminal flow rate increased). The porous stent without side holes showed similar mass flow rate profiles, although flow rate changes were more gradual in the proximal region of the ureter compared to the stent containing side holes. Moreover, both intraluminal and extraluminal flow rates continued to vary until about side hole 23, displaying a narrower plateau region compared to the stent with side holes.

## Discussion

Ureteric stents made of porous materials have gained increasing interest in recent years, since they provide additional functionalities compared to more conventional unporous stents, including enhanced biocompatibility, biodegradability, and drug loading capacity [29, 31, 54]. Despite several previous studies have been carried out to spatially resolve the flow dynamic field in the stented and occluded ureter [34, 36, 39, 41], to the best of the authors’ knowledge these earlier investigations have not reported on the flow performance of a porous stent. As discussed in greater detail in the remainder of this section, greater urinary drainage through the stented ureter and enhanced flow through a porous stent wall may have a range of beneficial effects. These include a potential reduction in encrustation rates and—in the case of biodegradable stents—a more gradual and spatially uniform degradation process. Therefore, to address the abovementioned research gap, in the present study a numerical model was employed to evaluate the flow dynamic field in the stented ureter, in the presence of a porous stent. This was achieved by solving for mass and momentum conservation equations over the designed computational domain (shown in Fig. 1), with the inclusion of a source term in the momentum conservation equation to account for the pressure gradient across the porous stent wall (as described by Darcy’s law).

It is well known that varying the permeability of a porous medium allows to modulate the transport of liquids through it [55], whereby permeability could be enhanced by increasing pore size and connectivity between pores, as previously reported for cross-linked polymer networks [56]. Therefore, in a first series of simulations, the effect of changing stent permeability on urinary drainage through the stented ureter was investigated (in the absence of a ureteral occlusion). Permeability values were varied in the range 10^−18^–10^−10^ m^2^, to model porous materials that are often employed to manufacture medical devices or scaffolds (such as collagen hydrogels, alginate hydrogels, and poly acrylamide gels). Results shown in Fig. 2 revealed—for the first time—that the total mass flow rate through the stented ureter and the fluid velocity within the stent (referred to as intraluminal velocity) increase non-linearly with increasing stent permeability. This observation could be attributed to the lower apparent hydraulic resistance offered by the stented ureter when a stent with greater permeability is employed, which in turn enhances urinary drainage through the ureter. Moreover, when the porous stent with the greatest permeability (10^−10^ m^2^) was employed instead of the unporous stent (see Fig. 3), it resulted in a 7.7% and 10.2% increase in the total mass flow rate through the stented ureter, in the absence and presence of a complete occlusion of the middle ureter respectively. These findings qualitatively corroborate with earlier studies postulating that ureteric stents made of porous materials (such as polylactic acid, degradable polyurethane and magnesium alloys, or other proprietary biodegradable materials) present similar or superior drainage performance compared to more conventional unporous stents [31, 57, 58]. Using a porcine model, Barros et al. showed that a proprietary hydrogel-based biodegradable stent caused an average grade of hydronephrosis (defined as the swelling of one or both kidneys) which was lower compared to an unporous commercial stent; this observation was attributed to improved drainage performance associated with the hydrogel-based stent [31]. Zhang et al. also showed reduced hydronephrosis after 2 weeks in a canine model, when a braided thin-walled biodegradable stent (containing side holes) was compared to an unporous stent [59]. These observations are consistent with the ones made by Chew and co-investigators on a porcine model, showing reduced hydronephrosis when a stent made of biodegradable copolymers was deployed in the unoccluded ureter instead of a conventional unporous inert stent [60]. Wang et al. observed comparable hydronephrosis between a porous poly(ε-caprolactone)/poly(lactide-co-glycolide) stent and a commercial unporous stent in a porcine model, up to 42 days. However, as the indwelling time increased further (beyond 56 days), the grade of hydronephrosis increased significantly for the unporous stent whilst it remained substantially unvaried for the porous stent [61]. Yin et al. carried out an in vitro study to investigate flow performance of a porous chitosan stent (without side holes and with anisotropic porous structure) compared to a commercial unporous stent containing side holes [54]. They quantified ‘effective viscosity’ as a measure of the stent’s ability to promote flow, and determined this parameter both on the stent alone as well as the stent inserted within a tube simulating the ureter. Results showed that effective viscosity was approximately 2.4 times greater in the porous stent compared to the unporous stent, when these were placed within the ureter model. However, it reduced of 48% (porous stent) and 31% (unporous stent) when stents were evaluated outside the ureter model. Unfortunately, the effect of stent wall permeability cannot be directly inferred from this study due to differences in geometry between stents (particularly concerning the presence of side holes). As a matter of fact, the lower effective viscosity for the unporous stent could be attributed to the presence of side holes. Upon removal from the ureter model, the improved drainage performance for the porous stent however corroborates its ability to effectively drain urine through the stent wall (even in the absence of side holes), which is consistent with results from the present study.

Notably, earlier studies have suggested that greater urinary flow through endourological devices reduces the extent and growth rate of encrustation, potentially increasing a device’s lifetime and reducing the occurrence of device-associated complications [62]. It could therefore be inferred that porous stents have the potential to offer greater device’s lifetime and improved safety. This appears to support earlier in vitro findings by Gorman et al., demonstrating superior resistance to encrustation and intraluminal blockage for a porous stent made of a poly(ethyleneoxide)/polyurethane composite hydrogel compared to unporous silicone and polyurethane stents (over a 24-week period) [63]. Fu and co-authors also observed calcifications on the surface of conventional polyurethane stents at 80 and 120 days from deployment in a canine model, whilst no calcification was observed on a porous stent made of poly-l-lactic acid and poly-dl-lactic acid [57]. Stents flow performance was however not evaluated in these previous investigations. Taking the findings from the present and previous studies all together, it would be recommended that further investigations are performed to evaluate porous materials with even greater permeability levels (i.e., > 10^−10^ m^2^), as these would promote urinary drainage to a greater extent. On the other hand, it is anticipated that reducing stent permeability further would not lead to significant changes in stent performance compared to the lowest value investigated (10^−18^ m^2^), and that only very limited fluid transfer would occur through the porous stent wall.

In a subsequent series of simulations, the distribution of urinary flow along the stented ureter was evaluated, by quantifying both intraluminal and extraluminal mass flow rates (as shown in Fig. 3). The flow distribution profiles for porous (permeability of 10^−10^ m^2^) and unporous stents were qualitatively comparable, and are also qualitatively consistent with the outcomes of previous CFD numerical studies by Kim et al. [38] and Mosayyebi et al. [36] on unporous ureteric stents. In the unoccluded ureter, urinary flow was predominately localised within the extraluminal compartment of the stent in the proximal region of the ureter. This is likely due to the greater hydraulic resistance offered by the stent lumen in comparison with the proximal ureter lumen. As the ureter cross-sectional area reduced along the proximal ureter towards the middle ureter, part of the urinary flow was directed into the stent resulting in a reduction of extraluminal flow rate and a corresponding increase of intraluminal flow rate, until the intraluminal flow exceeded the extraluminal one. Both flow rates subsequently plateaued to an almost constant value in the remaining sections of the middle and distal ureter, where the ureter cross-section remained approximately unchanged. As the stent entered the bladder compartment at the VUJ, the cross-sectional area of the urinary tract model increased and the fluid started to exit the stent, which was reflected in a corresponding increase of extraluminal flow rate and a reduction of intraluminal flow rate in this distal region. A complete occlusion of the middle ureter was then introduced within the model, as it represents one of the most recurrent causes of ureteral obstruction observed clinically [43]. The flow distribution profiles followed a similar trend as for the unoccluded ureter, with the exception of the region localised in close proximity to the obstruction. In this region, all extraluminal flow was directed within the stent to by-pass the obstruction, and a proportion of this flow subsequently exited the stent lumen post-obstruction. These results are coherent with those reported by Kim et al., who numerically simulated a stenosed ureter (with different degrees of stenosis) in the presence of an unporous double-J stent [39]. Overall, results confirm that the porous stent retains the ability to allow urinary drainage in the presence of a ureteral occlusion, as for a more conventional unporous stent, which supports previous in vivo studies evaluating the drainage performance of porous stents [31, 57, 58]. As discussed earlier, both intraluminal and extraluminal mass flow rate levels were greater for the porous stent compared to the unporous stent. This is likely due to the lower hydraulic resistance offered by the porous stent, where urine can flow both through the porous stent wall and the stent side holes. This provides an additional pathway for the fluid flowing through the stented ureter when compared to an unporous stent, for which fluid exchange can only occur through side holes.

These findings are further corroborated by those reported in Fig. 4, showing the mass flow rate through each individual side hole of the stent. Side holes that are interested by fluid transfer between intraluminal and extraluminal compartments are herein referred to as ‘active’, whilst side holes that are not interested by fluid transfer are referred to as ‘inactive’. In the unoccluded ureter, intercompartmental fluid exchange occurred predominately at side holes located in the proximal ureter and in the bladder compartment, as in these regions urine entered and exited the stent respectively. The remaining side holes were instead largely inactive. In the occluded ureter, side holes located just proximally and distally to the occlusion were also activated, allowing urine to by-pass the source of occlusion. Notably, the mass flow rate through active side holes of the unporous stent is almost twice that of side holes in the porous stent. This could be likely attributed to the additional pathways for fluid transport offered by a porous stent wall, which reduced the mass flow rate through side holes. However, in the unoccluded ureter, this also corresponded to a slight increment in the flow rate through side holes that were otherwise inactive in the unporous stent (i.e., from side hole 20 to 32). In previous studies using a microfluidic-based model of the stented ureter (referred to as ‘stent-on-a-chip’), it was demonstrated that inactive side holes suffer from accumulation of encrusting crystals [9, 64]. It is therefore anticipated that these differences in mass flow rate through side holes may impact on the initiation and growth of encrustation in a porous stent, and would thus merit further experimental investigations.

In a previous study using a full-scale physical model of the stented ureter, it was revealed that the cavity formed by a complete occlusion of the ureter lumen is characterised by the onset of low-velocity laminar vortices [42]. In subsequent studies using stent-on-a-chip models, it was further observed that this cavity flow promotes entrapment of both crystals and bacterial cells [9, 65]. In this study, direct fluid transfer through the wall of a porous ureteric stent was observed for the first time, including in the occluded region of the ureter (as shown in the velocity vector contours illustrated in Fig. 5). It is anticipated that fluid flowing through the stent wall may influence the characteristics of cavity flow in the occluded ureter, and this in turn may affect the progression of encrustation and bacterial deposition in this region of the ureter. Evidence of fluid flow through the stent wall can also have implications on the release mechanisms and kinetics of pharmaceutical compounds that may be loaded into the stent for antimicrobial, anticancer, or anti-inflammatory purposes.

In a final group of simulations, it was evaluated whether side holes play a critical function on drainage performance of a porous stent, considering its ability to also enable fluid transfer through the stent wall. According to a previous study by Kim et al., the intraluminal flow rate through an unporous stent without side holes remains substantially constant along the stent [38], as the fluid can only enter and exit the stent through its inlet and outlet cross-sections, respectively. In the present study however, it was demonstrated that a porous stent without side holes experiences variations in both intraluminal and extraluminal flow rates along the unoccluded ureter, which is due to the occurrence of fluid flow through the porous stent wall (see Fig. 6). The absence of side holes resulted in more gradual changes in mass flow rate, both intraluminal and extraluminal, compared to a porous stent with side holes. This is likely due to the fact that—in a porous stent—intercompartmental fluid transfer is distributed along the stent rather than concentrated only at discrete points (i.e. side holes). Moreover, whilst in the presence of side holes, changes in mass flow rate mainly occur in the proximal and distal segments of the stent, in the absence of side holes these appear to interest the middle ureter too (although to a lower extent). However, the presence of side holes overall resulted in greater total flow rate through the stented ureter, which further supports the utility of these stent’s features in promoting urinary drainage (even in a porous stent).

Changes in urinary drainage associated with a porous stent could also impact on stent degradation. This is particularly relevant for biodegradable stents, which have attracted increasing interest since they do not require removal through surgical intervention and are an effective strategy against the so-called ‘forgotten stent syndrome’. The degradation process of a porous material can be influenced by several factors, including fluid flow through the material, mechanical loading, and temperature of the surrounding environment [30, 66–68]. According to a previous study by Agrawal et al. on biodegradable scaffolds [30], the self-degradation products could also act as catalysers and accelerate the degradation process if they are not removed promptly from a scaffold. Under dynamic flow conditions, the autocatalytic degradation process could thus be inhibited by washing away the catalyser upon application of a fluid flow. In this respect, increasing the permeability of the degradable scaffold could result in lower degradation rates [30]. Considering the results from the present study, it could therefore be hypothesised that stents with greater permeability would present the lowest degradation rate, since they are associated with the highest intraluminal flow velocity. Moreover, it could be hypothesised that a porous stent without side holes may undergo a more spatially uniform degradation process, as flow variations along the stented ureter are less pronounced and more distributed in space compared to a stent containing side holes. However, these processes are dependent on the type of degradation mechanism and additional simulation- and experiment-based investigations should be carried out to evaluate the effect of flow dynamics on stent degradation more comprehensively. Overall, findings from this study suggest that maximising stent permeability would enhance drainage performance, potentially leading to increased stent lifetime and reduced side effects on patients. Since the porous stent wall allows for fluid transfer between intraluminal and extraluminal compartments, side holes may not be strictly required in a porous stent (although they further increase its drainage performance). In the case of a biodegradable stent, the absence of side holes may lead to more uniform stent degradation. However, given the ability of side holes to provide an effective path for urine to by-pass a source of ureteral obstruction, holes could be potentially manufactured only in the vicinity of the occlusion (i.e., on a patient-specific basis). The flow rate through side holes could also be increased, i.e. by varying the side hole size or shape, to minimise the risk of particle accumulation in these regions. The developed numerical model could thus be employed to guide the design of porous stents through optimisation of their material permeability, side hole location and size, thickness of the stent wall, inner diameter and other design characteristics. It should be noted that any identified porous stent configuration would require additional experimental verification; for instance, to assess whether it possesses sufficient mechanical strength for successful clinical deployment.

The model described in this paper builds on some assumptions and limitations; this paragraph elaborates on the potential implications of these. (a) The developed ureter model had a straight centreline, and both ureter and stent were axisymmetric. In a physiological scenario however, the ureter displays a number of patient-specific curvatures and strictures [45], and the stent may be in contact with the inner ureter wall in some regions. On the basis of earlier investigations [69], it could be inferred that the number of ‘active’ side holes may increase due to these physiological curvatures or asymmetries, because of local fluid pressure gradients established between the stent and ureteral lumens. These pressure differences may also locally enhance fluid flow through a porous stent wall. (b) The pressure difference between kidney pelvis and bladder was assumed constant and the ureter wall was assumed rigid. This implies neglecting the peristaltic activity of the ureter, as well as the periodic priming and voiding of the bladder (known as micturition). Previous studies have shown that the deployment of a ureteric stent causes a notable inhibition of ureteral peristalsis [50], which supports the assumption of stationary flow in the present and previous modelling studies. A time-dependent model could however be developed to simulate bladder micturition, which would allow recapitulating the onset of retrograde flow from the bladder towards the kidney pelvis during bladder contraction (known as reflux). It may be anticipated that varying the stent permeability could influence the magnitude of retrograde flow, consistently with the observations from the present study concerning anterograde flow. (c) The structural properties (i.e. elasticity and compliance) of ureter and stent were not modelled. A previous fluid–structure interaction (FSI) study has shown that there is negligible mechanical interaction between the stented ureter and urine, for stents made of common unporous materials [70]. Whether these observations apply to porous stents has yet to be established. Porous stents made of softer and more compliant materials may potentially undergo radial changes as a result of local pressure variations or upon compression caused by kidney stones, tumours or strictures. These changes may potentially impact on stent permeability locally, for instance by altering the pore size in the compressed or dilated region. (d) The porous stent wall was assumed isotropic and homogenous. An earlier microstructural analysis of a porous chitosan stent has revealed an anisotropic architecture with multidomain texture [54]. If, for example, pore size was to vary from the inner to the outer surface of the stent wall, this would impact on the proportion of fluid flow directed into the stent compared to the one exiting the stent. Yin et al. hypothesised that a decrease in pore diameter from the outer to the inner stent surface may increase flow within the stent lumen, due to capillary effects [54]. The pores architecture would however be highly dependent on the stent material and manufacturing method adopted. (e) Ureteral occlusions can have a range of different shapes and dimensions [71]. In this study, a fillet was not built into the model at the occluded region. Varying the shape of occlusion may potentially impact on the characteristics of cavity flow and the fluid velocity magnitude through the porous stent in the occluded region of the model. Finally, (f) urine physical properties (i.e., density and viscosity) may be affected by certain pathological conditions, such as urinary tract infections. For instance, previous studies have postulated on increased urine viscosity associated with infectious conditions [42]. This would result in a reduction of fluid velocity magnitude throughout the stented ureter (and through the porous stent wall), although the flow distribution patterns are likely to remain largely unaffected.

## Conclusion

Porous ureteric stents have attracted considerable interest because of their biocompatibility, biodegradation potential and drug loading capacity. However, characterisation of their flow dynamic performance is very limited, which may hinder further technological developments and translational research in this area. To the best of the authors’ knowledge, this study is the first to report on a numerical analysis of the fluid dynamic field in a porous ureteric stent within a model of the urinary tract. A model of ureteric stent was reconstructed from a commercially available double-J architecture, and coupled with models of both the unobstructed and obstructed ureter (with the obstruction located in the middle ureter). Stent permeability was varied in the range 10^−18^ to 10^−10^ m^2^, to mimic porous materials that are often employed in biomedical devices or scaffolds. Results revealed that urinary drainage through the unoccluded stented ureter increases non-linearly with increasing stent permeability, suggesting that a porous stent could offer improved drainage performance compared to its unporous counterpart. In the unoccluded ureter, the total mass flow rate increased of 7.7% when a porous stent with permeability of 10^−10^ m^2^ was employed instead of an unporous stent. A further improvement in drainage performance was observed in the presence of a ureteral occlusion, with the porous stent resulting in 10.2% greater mass flow rate. The flow distribution within the stented ureter was qualitatively similar between porous and unporous stents, in both unoccluded and occluded ureters, which further corroborates the ability of a porous stent to maintain urinary drainage in the presence of a complete occlusion of the ureter lumen. The presence of a porous stent wall caused a reduction in mass flow rate through side holes, in both occluded and unoccluded ureters, as urine could flow both through the stent wall and side holes of the porous stent. However, in the occluded ureter in particular, this also corresponded to an increase in the number of ‘active’ side holes compared to the unporous stent, which could have potential beneficial effects in preventing or reducing encrustation at side holes. Results also provide evidence of fluid flow through the stent wall, which could potentially impact on the onset of cavity flow within the occluded region of the ureter, as well as modulate drug release kinetics in the case of a drug-eluting stent. Finally, removal of side holes from a porous stent resulted in more gradual variations in extraluminal and intraluminal flow rates along the stent, which are indicative of fluid transfer occurring through the stent wall. This however was accompanied by a reduction in the overall drainage performance of the stent. Findings from this study provide some fundamental insights into the flow dynamic performance of porous ureteric stents, with potential utility in the development pipeline of these medical devices. Future studies could address some of the limitations of the model presented in this paper, such as the isotropy of the porous material, the static nature of the ureter and bladder walls, and the lack of physiological curvatures in the ureter model. A wider range of conditions could also be simulated to represent distinct patient orientations and locations. A future model could also incorporate biodegradation and drug elution, to further inform optimisation of these processes. Experimental studies could also be carried out to directly and quantitatively correlate the flow performance of a porous ureteric stent with its degradation and/or drug elution behaviour. Notably, there is a lack of experimental studies that quantitatively evaluate flow performance of porous ureteric stents. Building on previous research [9], a microfluidic-based device replicating features of the stented ureter could be developed to quantify flow through porous stents of varying permeability and to correlate flow performance with the rate of stent degradation. This approach would also potentially enable an experimental validation of the numerical model framework developed in this study.

## Supplementary Information

Below is the link to the electronic supplementary material.

Supplementary file1 (DOCX 436 KB)
