# Supplementary material for: Computational simulation of the flow dynamic field in a porous ureteric stent
Source: Med Biol Eng Comput. 2022 Jun 28;60(8):2373–87. doi: 10.1007/s11517-022-02620-1 (PMC9294020; doi:10.1007/s11517-022-02620-1)
Supplement: Supplementary file 1 — Supplementary file1 (DOCX 436 KB) [file 11517_2022_2620_MOESM1_ESM.docx]

**Supplementary Material for:**

**Computational simulation of the flow dynamic field in a porous ureteral stent**

Xiaohan Yang^1^, Ali Mosayyebi^1^, Dario Carugo^2^

^1^ Department of Mechanical Engineering, Faculty of Engineering and Physical Sciences, University of Southampton, Southampton, UK

^2^ Department of Pharmaceutics, UCL School of Pharmacy, University College London, London, UK

Corresponding author: Dr. Dario Carugo, [d.carugo@ucl.ac.uk](mailto:d.carugo@ucl.ac.uk), +442039872803


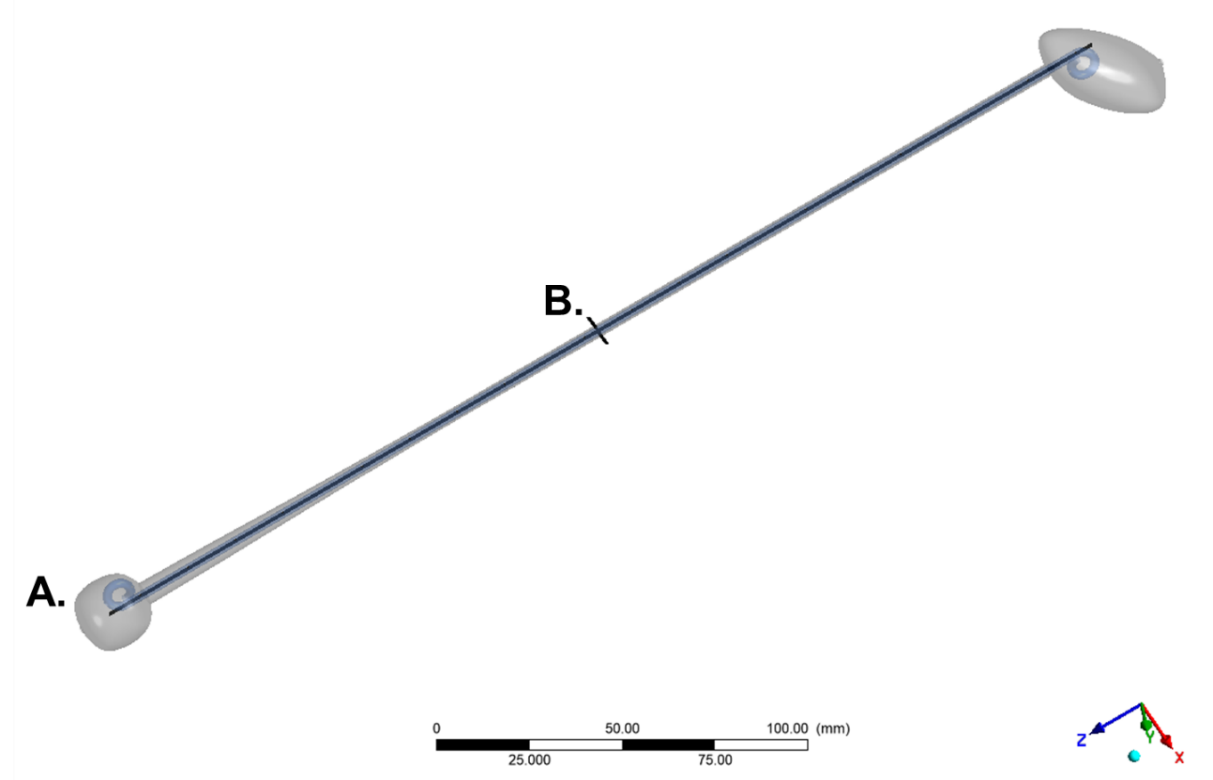


**Fig. S1** Graphical representation of the stented ureter model, showing the two lines along which values of fluid velocity magnitude and static pressure were determined for mesh sensitivity analysis. Line A corresponds to the centreline of the stented urinary tract (in the same direction of z), along which static pressure was determined. Line B instead corresponds to a line perpendicular to line A, in the same direction of x, along which the fluid velocity magnitude was determined. Line A was located in the model mid-plane, and in the middle region of the ureter.


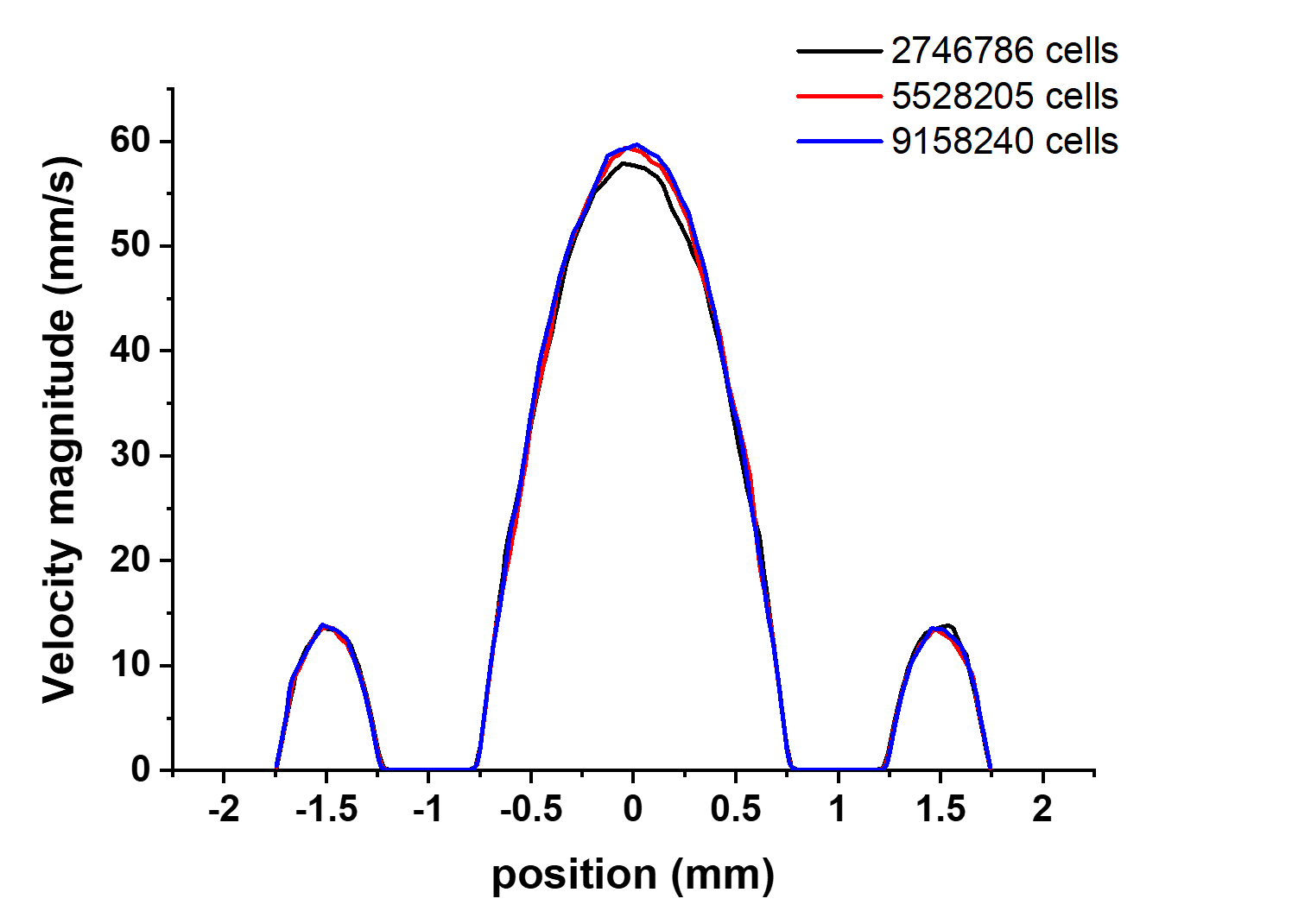


**Fig. S2** The plot of fluid velocity magnitude (in mm/s) along line B, for the three different mesh densities evaluated in the mesh sensitivity analysis. These corresponded to a total of 2 746 786 (black line), 5 528 205 (red line), and 9 158 240 (black line) cells, respectively.


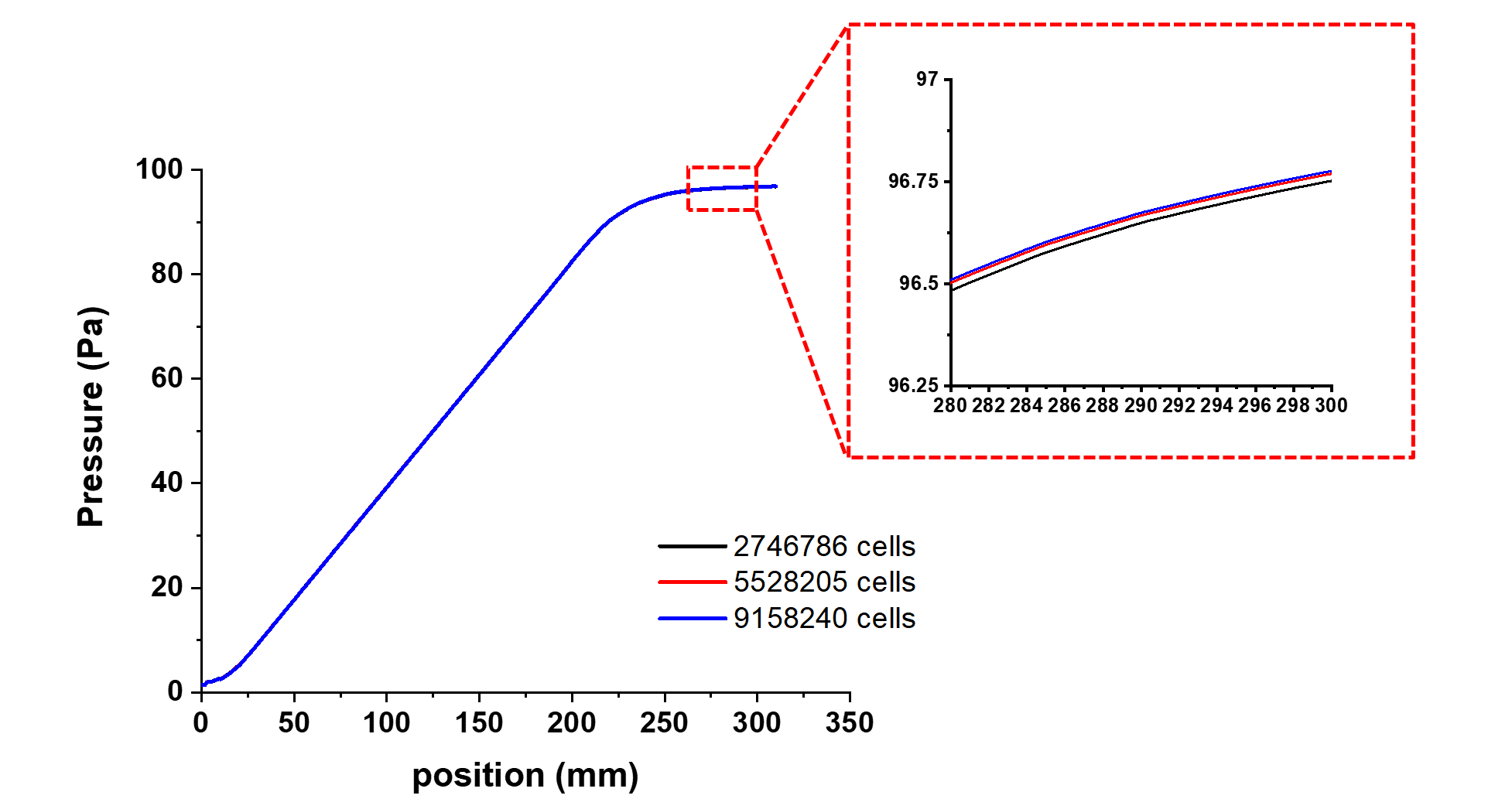


**Fig. S3** The plot of fluid static pressure (in Pa) along the centreline of the ureter model (line A), from the kidney pelvis (right) to the bladder (left) compartments, for the three different mesh densities evaluated in the mesh sensitivity analysis. These corresponded to a total of 2 746 786 (black line), 5 528 205 (red line), and 9 158 240 (black line) cells, respectively. The red dashed box includes a zoomed-in view of the plots, in the region of the kidney pelvis, to more clearly illustrate differences between curves.

**
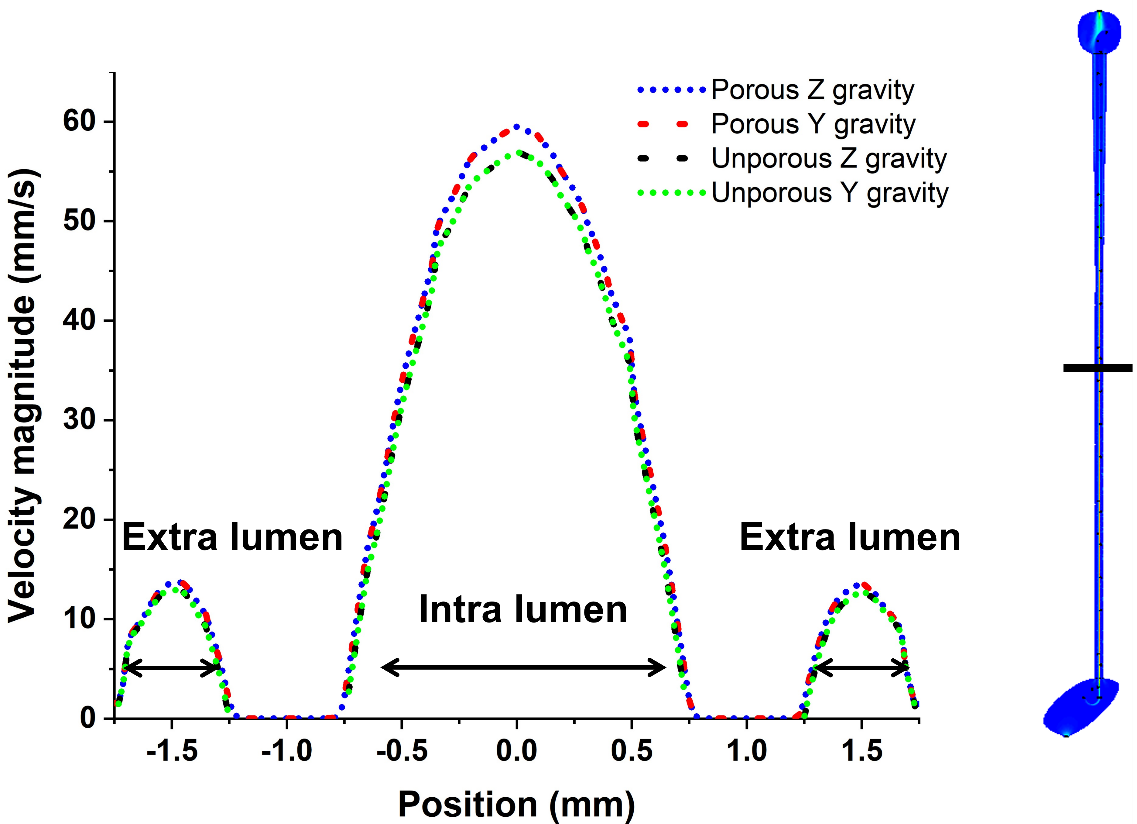
**

**Fig. S4** a. Profile of velocity magnitude (in mm/s) taken along a line located in the mid-ureter, for different orientations of the stented ureter model. These corresponded to a patient either in supine (Y gravity) or standing (Z gravity) position. Both porous (permeability: 10^-10^ m^2^) and unporous stents were modelled. The model schematic on the right-hand side shows the location of the line along which the velocity values were taken. Intraluminal and extraluminal compartments are also labelled on the graph.
